# The optimal sampling design for littoral habitats modelling: A case study from the north-western Mediterranean

**DOI:** 10.1371/journal.pone.0197234

**Published:** 2018-05-24

**Authors:** Maria Elena Cefalì, Enric Ballesteros, Joan Lluís Riera, Eglantine Chappuis, Marc Terradas, Simone Mariani, Emma Cebrian

**Affiliations:** 1 Centre d’Estudis Avançats de Blanes-CSIC, Acc. Cala Sant Francesc, Girona, Spain; 2 Estación de Investigación Jaume Ferrer, Instituto Español de Oceanografía (IEO), Mahón, Spain; 3 Departament de Biologia Evolutiva, Ecologia i CiènciesAmbientals, Facultat de Biologia, Universitat de Barcelona, Gran Via de les Corts Catalanes, Spain; 4 Departament de Ciències del Mar i Biologia Aplicada, Universitat d’Alacant, Apartat de Correus, Spain; 5 Institut d’Ecologia Aquàtica, Universitat de Girona, Plaça Sant Domènec, Spain; University of Waikato, NEW ZEALAND

## Abstract

Species distribution models (SDMs) have been used to predict potential distributions of habitats and to model the effects of environmental changes. Despite their usefulness, currently there is no standardized sampling strategy that provides suitable and sufficiently representative predictive models for littoral marine benthic habitats. Here we aim to establish the best performing and most cost-effective sample design to predict the distribution of littoral habitats in unexplored areas. We also study how environmental variability, sample size, and habitat prevalence may influence the accuracy and performance of spatial predictions. For first time, a large database of littoral habitats (16,098 points over 562,895 km of coastline) is used to build up, evaluate, and validate logistic predictive models according to a variety of sampling strategies. A regularly interspaced strategy with a sample of 20% of the coastline provided the best compromise between usefulness (in terms of sampling cost and effort) and accuracy. However, model performance was strongly depen upon habitat characteristics. The proposed sampling strategy may help to predict the presence or absence of target species or habitats thus improving extensive cartographies, detect high biodiversity areas, and, lastly, develop (the best) environmental management plans, especially in littoral environments.

## Introduction

The prediction of species and habitat distributions through numerical models still represents one of the most challenging areas of work in ecology [1], especially in light of the current scenario of a rapidly changing environment. Species distribution models (SDMs) and habitat distribution models (HDMs) find many applications in ecology [2, 3], including conservation and management [4], and, in conjunction with rich, high coverage data sets and simulation experiments, may help in designing efficient sampling strategies for habitat suitability modelling [5] for both terrestrial and marine areas.

SDMs and HDMs are statistical models of the relationship between species and habitat distributions, and those environmental variables that potentially drive such distributions [2]. Mechanistic, empirical (or correlative), and theoretical models can be used, depending on the research objectives and the variables available [2]. Empirical models are most frequently used, especially those coupling the distributions of species and environmental variables [6]. Recent access to data from remote sensing techniques and geomorphological cartographies, as well as rapid advances in geographical information systems (GIS) have provided large sets of species and environmental data to build empirical models [1]. Empirical models relate known occurrences (presence and absence) of species or habitats to the environmental variables that best reflect the species’ or habitats’ environmental requirements. Once the empirical model is fitted, the output is used to predict the most suitable or unsuitable areas for species and habitats [7].

The degree to which causal relationships between species/habitat distributions and the predictor variables are unveiled depends on the adequacy of the predictors used for model building and on the quality of species or habitats occurrence data [8, 9]. Sample size, sample design, species and habitat characteristics, environmental stratification, and species prevalence are also important for the success of predictive spatial distribution models [2, 6, 8, 10, 11]. The paucity of fine environmental and species occurrence data for marine ecosystems may explain why sea-focused SDMs and HDMs are fewer compared to terrestrial ones [12]. However, many efforts have been made in recent years to collect data on environmental variables and species distributions (e.g. BIO-ORACLE [13], OBIS, www.iobis.org), thus allowing the application of SDMs to marine systems. Most models aim to predict the potential distribution of one or a few benthic species or habitats of special conservation interest [14–19], endangered fish species that are commercially exploited [20–22], or the effects of global change on a single species or habitat [23–27]. Nevertheless, most researchers have not yet analyzed the importance of sample size, sampling design, or species occurrences to build up accurate SDMs for marine environments. Those parameters are crucial for achieving the best accuracy (as measured by AUC, area under the receiver operating characteristic [ROC] curve) and performance (measured as sensitivity and specificity) in predictive SDMs [5].

The littoral zone harbors a rich array of habitats [28–30] with specific environmental requirements. Habitat cartographies require much detail to cope with the small-scale variability of littoral habitats and species distributions. This variability requires big, often expensive sampling efforts. It is paramount then, to define valid, logistically easy-to-perform and competitive sampling strategies to achieve species distribution models for large spatial areas. Additionally, littoral habitats are often exposed to many environmental pressures and disturbances [31]. Monitoring possible changes in habitat distribution patterns, especially in relation to anthropogenic pressures may help improve both local and international management actions and build up new bioindicators to be used in Habitat Directives. In Europe, for instance, this is critical to conservation actions for the Habitats Directive (92/43/EC) and the Marine Strategy Framework Directive (2008/56/EC) and thus this study reinforces the validity of these habitats as working units. Hence, there is an increasing need to investigate the extent of the relationships between species, habitats, and environmental pressures to obtain models that predict with the maximum accuracy and performance littoral habitat shifts in response to environmental changes [32, 33].

Recently, Cefalì et al. [34] analyzed the relationship between littoral habitats and environmental factors from a large, high-resolution dataset (16,098 data points), identifying the environmental variables associated with the spatial distributions from a total of 29 littoral habitats. In this paper, we use this dataset, which integrates the occurrence (presence and absence) of rocky littoral habitats and that of environmental variables such as shore slope, geology, wave exposure, seawater temperature, and substrate type, [34, 35] to build HDMs for a long (562,895 km) stretch of rocky coastline. Specifically, in this study, we explored the relevance of sampling design and sample size to the accuracy and performance of predictive models. Our aim was to assess the best sampling strategy to predict the distribution of coastal habitats with a resolution of tens of meters. The specific objectives of this study are: 1) to identify the best (in terms of accuracy, performance, and cost-effectiveness) sampling strategy and sample size for building predictive models for six rocky littoral habitats and to produce predictive maps of potential habitat distribution at a regional scale; 2) to assess changes in model accuracy and performance for habitats with different distributional patterns (i.e. abundant and widely distributed, abundant and locally distributed, uncommon habitats); and 3) to examine how sample size, sample design, habitat characteristics, and habitat prevalence (occurrence, frequency) may influence model accuracy and performance.

## Material and methods

### Ethics statements

The permission for the field studies and especially for the MPAs (Illes Medes, Montgrí, and S’Encalladora Marine Reserve and the National Park of Cap de Creu) was provided by the Catalonia Government. This study is based on observational data and no animal or algae, endangered or protected species were collected.

### Study site

**Fig 1:**
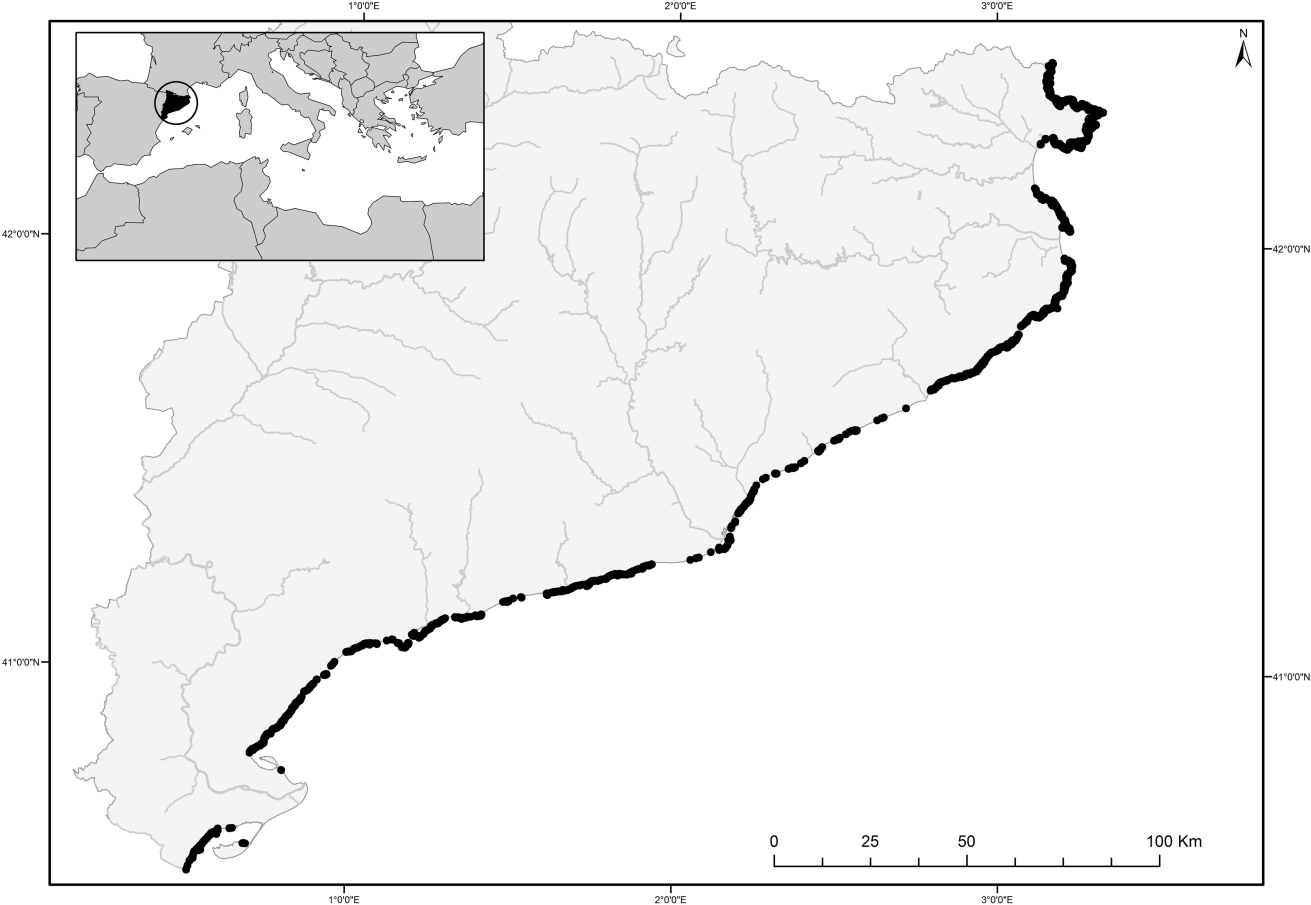
Map of the study site. The 16,098 points along the coast contain information on habitats presence/absence and values of environmental variables. The map was created with ArcGis 10.1 (ESRI).

Data on littoral habitat distribution and environmental variables were collected along the whole coast of Catalonia (North-Western Mediterranean between 3º 10' 28.072" E, 42º 26' 17.619" N and 0º 30' 57.001" E, 40º 31' 26.302" N) (Fig 1). This coast shows high geomorphological heterogeneity [36, 37], a very complex tectonic setting [38], and strong differences in the geometry of the coastline from north to south. The northern coast is in fact much more irregular than the central and southern ones. The studied coast encompasses most of the Mediterranean rocky littoral habitat diversity, including natural and artificial (man-made) hard-bottom environments [35]. Sampling was done by recording the presence of all habitats from the supralittoral to the upper infralittoral level (-1 m a.m.s.l.). More details about the sampling and dataset generation for this exhaustive habitat cartography are in Mariani et al. [35] and Cefalì et al. [34]. The original database is a layer of 16,098 points with biological (habitat presence) and environmental information (Fig 1) and covers the complete rocky coastline (562,895 km) of Catalonia (10 m resolution). Habitats are defined following the definition of the European Habitats Directive (92/43/EEC) and named by the dominant species. This exhaustive cartography of the littoral habitats is available online for the entire Catalan coast from (http://mediambient.gencat.cat/es/05_ambits_dactuacio/patrimoni_natural/sistemes_dinformacio/habitats/habitats-litorals-/).

### Data collection

We have modeled the spatial distribution of six littoral habitats (Table 1), which were strongly and significantly influenced by environmental variables [34]. These habitats showed different distributional patterns, although most of them had clear north distributions. The habitats of the red alga *Rissoella verruculosa* and the crusts (no rim-forming) of the coralline alga *Lithophyllum byssoides* were also abundant but nearly absent in the south [39]. The rim-forming *Lithophyllum byssoides* (so-called “Trottoir”, present in the northern coast) and the *Neogoniolithon brassica-florida* concretions (present in the south) were overall uncommon and localized [39]. Finally, the habitat of the cave-dwelling red algae *Hildenbrandia rubra* and *Phymatolithon lenormandii* showed a very scattered distribution along the coast [39]. The habitat dominated by the brown alga *Cystoseira mediterranea* [39–42] was overall abundant and widespread.

**Table 1:** List of the habitats studied. Number of occurrences and frequency (F) of selected habitats in the original database (16,098) are presented. Each habitat is named after the principal species that characterizes it. Habitat characteristics are from Ballesteros et al. [39].

Habitat	N	F (%)	Habitat characteristics
*Rissoella verruculosa*	7710	47.9	Mediolittoral habitat from exposed littoral environments, preferably on plutonic rocks dominated by the red alga *R*. *verruculosa*.
*Lithophyllum byssoides*	5621	34.9	Mediolittoral habitat from environments with high desiccation levels and strong hydrodynamism dominated by the red coralline *L*. *byssoides*.
*Lithophyllum byssoides* rims ("Trottoir")	1154	7.2	Characteristic rims of the red coralline *L*. *byssoides* on very exposed, mediolittoral rock with low irradiance, preferably on calcareous or metamorphic rock.
*Neogoniolithon brassica-florida*	528	2.8	Association with the red coralline *N*. *brassica-florida* and/or the mollusc *D*. *petraeum* on moderately-to-calm mediolittoral rocks.
*Hildenbrandia rubra/* *Phymatolithon lenormandii*	119	0.7	Mediolittoral caves and overhangs.
*Cystoseira mediterranea*	4576	28.4	Shallow, exposed and well-lit infralittoral rock dominated by the brown alga *C*. *mediterranea*.

The distribution of all habitats (Table 1) was significantly influenced by environmental variables [34]. The variables used as predictors were (Table 2): minimum and mean wave height (WH, data from 1998 to 2008), estimated using the Downscaled Ocean Waves model (DOW) [43]; mean sea surface temperature (SST; data from 2003 to 2010), obtained from satellite measurements performed by the MODIS (aqua) sensor system (http://oceancolor.gsfc.nasa.gov/), available as “Ocean Level-2” HDF data by NASA's Goddard Space Flight Center; rock slope, obtained from a Digital Elevation Model (DEM) created with a LiDAR detection method by the Institut Geològic i Cartogràfic de Catalunya (IGCC); rock geology (plutonic, sedimentary, metamorphic and mineral), provided by the Institut Geològicic i Cartogràfic de Catalunya (IGCC, www.igc.cat); finally, the substrate type (an index of two categories identifying whether the rocky substrate was natural or man-made), obtained from the CARLIT data set [37]. As detailed in Cefalì et al. [34], spatial resolution grain sizes were: 0.01º latitude and 0.008º longitude for minimum and mean wave height; a data point every 10 km for mean sea surface temperature; a raster format with pixel resolution of 2 x 2m for rock slope; a 1:50.000 map scale for rock geology and a map scale of 1:1000 for substrate type.

**Table 2:** List and description of the environmental variables studied. A detailed explanation on the variable source and the calculation method are provided in the text.

Environmental predictors	Units or Categories	Source	Year
Average Sea Surface Temperature	16.8º – 18.7º °C	MODIS	2003–2013
Average Wave Height	0.02–0.9 m	DOW	1998–2008
Minimum Wave Height	0.01–0.07 m	DOW	1998–2008
Slope	0º – 10.8º	DEM	2014
	10.8º – 22.8º	DEM	2014
	22.8º – 45.1º	DEM	2014
	45.1º – 68.2º	DEM	2014
	68.2º – 87.8º	DEM	2014
Geology	Metamorphic	IGCC	2000
	Mineral	IGCC	2000
	Plutonic	IGCC	2000
	Sedimentary	IGCC	2000
	Artificial	IGCC	2000
Substrate type	Natural	CARLIT	2012
	Artificial	CARLIT	2012

### Sampling scenarios

**Fig 2:**
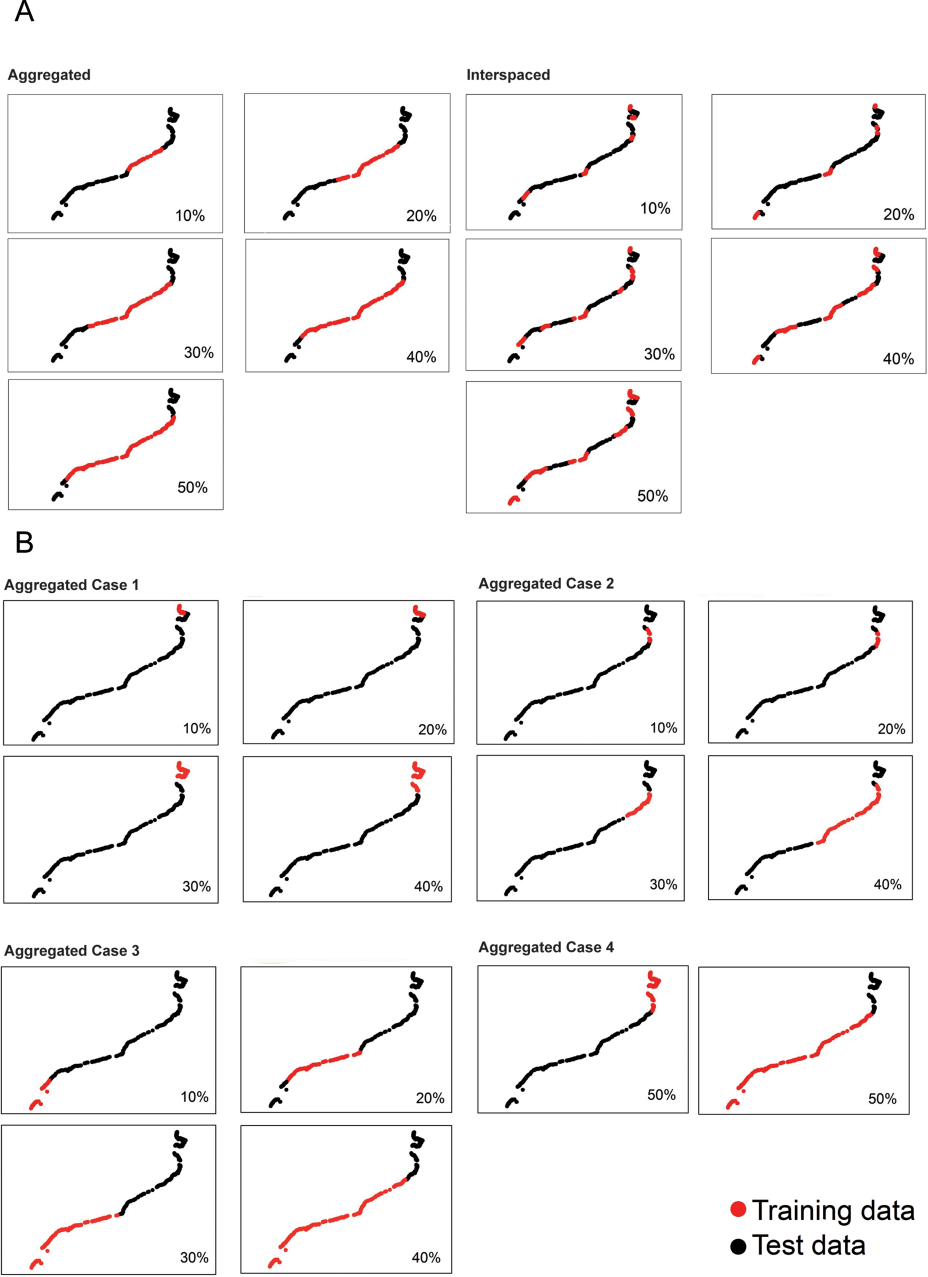
Sampling designs. Sampling scenarios considered in this study based on combinations of sampling size (20%, 30%, 40%, 50%) and sampling strategy: A) aggregated and interspaced; B) aggregated case 1, case 2, case 3 and case 4. Red dots represent data points selected for model training whereas black dots represent data points used for model validation. Random samples for null models are not depicted. Please notice that the percent sampling size (either red or black dots) may appear unrealistic at the scale of the figure. This is because of the extremely irregular shape of the northern portion of coast where much more data points exist.

Two strategies for data sampling were compared to address the first objective of the study, aggregated and interspaced. For the aggregated sampling strategy, a unique stretch of arbitrarily chosen neighboring points was selected (Fig 2). For the interspaced sampling, we selected a minimum of 5 data units (stretches of continuous points) interspaced by equivalent numbers of unselected data points (Fig 2). To assess changes in model accuracy and performance for habitats with different distributional patterns (see above), different scenarios were performed for the aggregated samplings considering different spatial distributions from north to south (Fig 2). The interspaced sampling inherently gathered data from the whole coast.

To define the best cost effective sampling size, we tested 5 different sampling sizes from the original matrix (16,098 points; 562,895 km): 10% (1,610 points; 56,290 km), 20% (3,219 points; 112,579 km), 30% (4,829 points; 168,868 km), 40% (6,439 points; 225,158 km), and 50% (8,049 points; 281,447 km) and for both, the aggregated and interspaced sampling (Fig 2). We compared the models performed with both aggregated and interspaced scenarios and different sampling sizes. Additionally, null models were fitted to randomly selected points for each sampling size (from 10% to 50%). All scenarios were applied to each of the 6 selected habitats. All spatial selections were performed in ArcGIS 10.1 (ESRI), whereas the random sampling for null models was made in R (R Development Core Team 2011).

### Habitat modelling

Since our habitat data were binary, to describe the relationship between the distribution of habitats and environmental variables (Table 2) we fitted generalized linear models with binomial error distribution and the logistic link function (GLM, [44]) using the entire dataset (16,098 points). The most parsimonious model for each habitat was obtained through variable selection using the “glmulti” function in the glmulti R package [45] based on AIC values. The environmental variables selected for each of the habitats are listed in Table 3. Samples, selected as described in the previous section, were used as training datasets to build the models. The remaining data were used as test data for model validation. For example, in Fig 2, the 10% portion of the coast sampled was used as training dataset and the remaining 90% was used as test dataset. The same procedure was repeated to compare the performance of all models built for each of the sampling scenarios. Model fit was assessed as the proportion (%) of explained deviance (D^2^):

$$D^{2}=\frac{\left(null\;deviance-residual\;deviance\right)}{null\;deviance}\times \;100$$

**Table 3:** Principal results. The most important environmental predictors, the best cost-effective models, the frequency (F %) of habitat in each sampling strategy and the model prediction results are shown. For more information about relationships between predictors and habitats, see Cefalì et al. [34].

	Principal environmental predictors	Best cost effective model strategies	Habitat F (%)	AUC	threshold	se	spe
*R*. *verruculosa*	SST average WH average Slope Geology Substrate type	20% interspaced	0,4936	0,87	0,57	0,90	0,74
*L*. *byssoides*	SST average WH average WH minimum Slope Geology	20% interspaced	0,3988	0,77	0,40	0,92	0,54
*L*. *byssoides* rims	SST average WH average WH minimum Slope Geology	10% interspaced	0,160	0,87	0,18	0,74	0,86
		20% interspaced	0,031	0,75	0,03	0,74	0,68
*N*. *brassica-florida*	SST average WH average WH minimum Slope	30% aggregated	0,01	0,90	0,21	0,94	0,87
		20% interspaced	0,399	0,77	0,4	0,92	0,54
*Hildenbrandia / Phymatholiton*	SST average WH average Geology	20% interspaced	0,0037	0,73	0,01	0,37	0,90
		30% interspaced	0,0033	0,81	0,01	0,65	0,79
		30% aggregated case 2	0,0161	0,82	0,02	0,74	0,81
*C*. *mediterranea*	SST average WH average WH minimum Slope Geology Substrate type	20% interspaced	0,295	0,77	0,37	0,84	0,61

Altogether, we fitted 19 models for each of the 6 selected habitats. The same procedure was conducted for the null models, where each random selection (10%, 20%, 30%, 40%, and 50%) was used as training data and tested on the remaining data, and repeated 10 times. For the null models, the D^2^ value presented is the mean and standard deviation of the 10 fitted models. All statistical analyses were performed in R (R Development Core Team 2011).

### Model validation

Model selection based on AIC identifies the “best” model among the set of candidate models, but it does not measure its performance in predicting independent data. To assess the predictive accuracy and performance of our models, we employed three statistics that compare the predictions to the observations in the test data: AUC (area under the receiver operating characteristic [ROC] curve), sensitivity (se), and specificity (spe). Because binomial GLM predictions are continuous probabilities between 0 and 1, we must specify a cut-off threshold to convert the continuous predictor to a discrete, binary predictor in order to calculate the percentage of correct classifications [10]. AUC is a synthetic index of the model accuracy, and is independent of threshold choice, weighing omission, and commission errors equally [10, 46, 47]. We used the AUC, which ranges from 0 to 1, as first model selection, where values ≤ 0.5 indicating that the model had not predictive power and 1 meaning that we had a good model. Following Swets [48], models providing values > 0.9 were considered “highly accurate”, those providing values in the range 0.7–0.9 were considered “useful”, and those with AUC below 0.7 are “poorly accurate”.

However, to select the best models and their discrimination power, it is necessary to calculate the percentage of predicted versus observed presences and absences. For this purpose, sensitivity and specificity were derived from a confusion matrix. Sensitivity (or true positive rate) is the portion of data points for which presence was correctly predicted, whereas specificity (true negative rate) is the portion of data points for which absence was correctly predicted [10]. Because habitats differed in their prevalence, we decided to use habitat-specific classification thresholds that maximized the sum of sensitivity and specificity [49]. Both sensitivity and specificity range from 0 when the model is completely inaccurate to 1 when either presences or absences are well predicted [50, 51]. The mean AUC, sensitivity and specificity of the 10 null models were also calculated. Analysis of AUC, and sensitivity specificity were performed in R (R Development Core Team 2011), using the pROC [52] and SDMTools [53] packages respectively.

## Results

### Best model strategy

Model accuracy and performance in predicting the distribution of the six benthic littoral habitats were tested for sampling design, sample size, and habitat prevalence. Only models with high accuracy (AUC > 0.70) and performance (sensitivity and specificity > 0.60) were considered.

**Fig 3:**
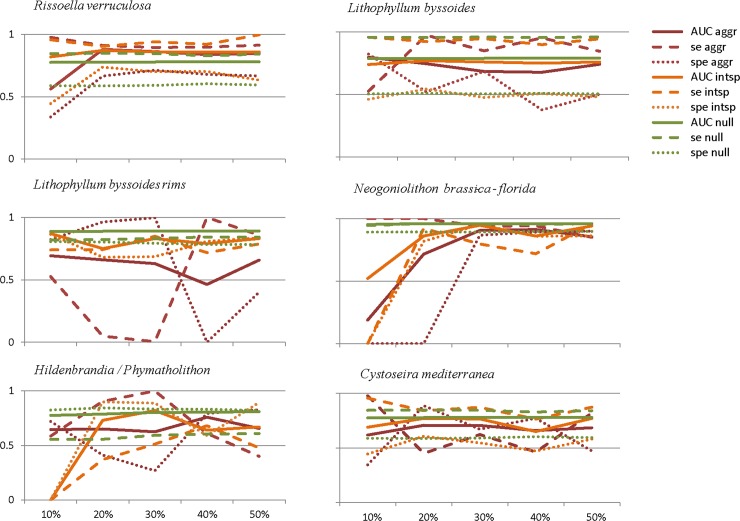
Statistical diagnostics of the predictive models for the aggregated strategy (aggr), the interspaced strategy (intsp) and the null (null) models. In each panel, the x-axis shows the sample size for the training data set (Fig 2). The y-axis, with values from 0 to 1, shows AUC, sensitivity (se), and specificity (spe) for each of the three sampling strategies.

As expected, null models were the most accurate and had the highest performance values (high AUC, sensitivity and specificity values) (Fig 3), and provided performance standards against which to compare the other sampling strategies. Although model performance was habitat-dependent, in general it was strongly dependent on the sampling design, with the best strategy being the interspaced data collection. The prediction performed with the interspaced strategy obtained accuracy and performance values close to the null models for all habitats studied, regardless the habitat spatial distribution (Fig 3, Table 3, S1 File).

In contrast, the results of aggregated sampling designs depended on the prevalence of the habitat considered. In fact, aggregated strategies performed well where habitat prevalence was high (i.e. *Rissoella verruculosa*, *Lithophyllum byssoides*, *Cystoseira mediterranea*) or with large sampling size (i.e. *Hildenbrandia/Phymatholiton* and *Neogoniolithon brassica-florida*). In contrast, habitat sample size and spatial distribution had lower effect on model accuracy and performance with the interspaced strategy. In fact, with the interspaced sampling and only 20% sampling size, we reached sufficient prevalence to obtain good model predictions for all the habitats considered. In general, with the interspaced design, increasing sample size did not substantially increase model accuracy and performance (Fig 3, Table 3, Tables A-F in S1 File). These results agreed with the null models, where increasing sample size did not always result in increased accuracy and performance prediction (Table 3, Tables A-F in S1 File).

### Predictive habitat models

**Fig 4:**
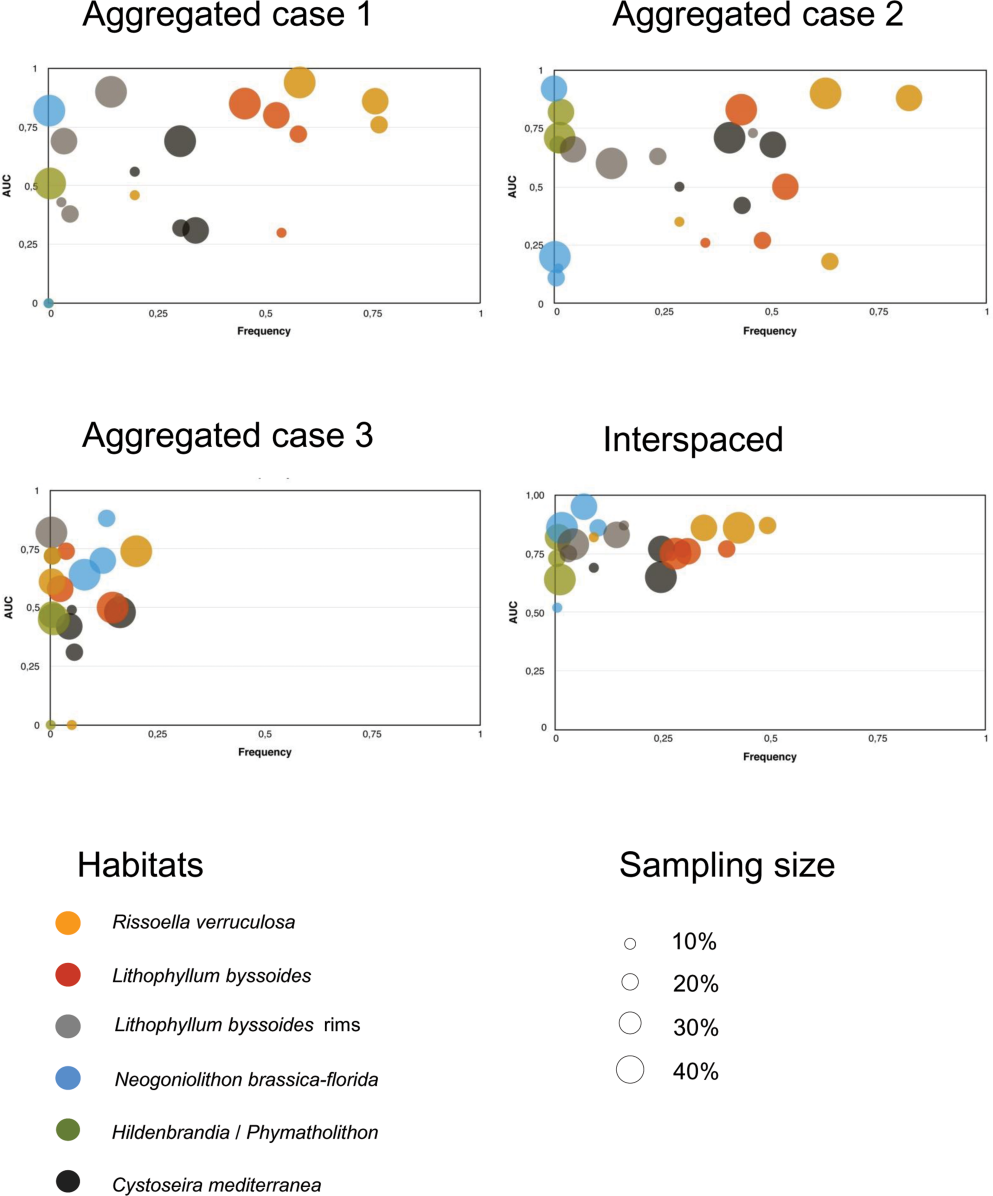
Habitat frequency and AUC values. Relation between habitat frequency (x-axis) and AUC values (y-axis), for sample sizes from 10% to 40% are presented.

Model performance was clearly habitat-dependent. Models for abundant but localized habitats (*Rissoella verruculosa* and *Lithophyllum byssoides*) were in general highly accurate and showed good performance (high specificity and sensitivity), with values comparable to those of null models (AUC > 0.80 for both habitats) (Table A and Table B in S1 File). The interspaced design provided the best model predictions (Fig 4). With 20% sample size we obtained models with good accuracy and performance for *R*. *verruculosa* (AUC = 0.87, sensitivity = 0.90 and specificity = 0.73) and for *L*. *byssoides* (AUC = 0.77, sensitivity = 0.92 and specificity = 0.54). In the interspaced design, increasing habitat occurrence improved model accuracy and prediction performance independently of sample size. With the aggregated designs, accurate and good performance models were obtained only with large sample sizes, regardless of habitat occurrence (Fig 4, Table 3, Table A and Table B in S1 File).

For uncommon and localized habitats (i.e. *Lithophyllum byssoides* rims and *Neogoniolithon brassica-florida* concretions), model predictions were accurate and performed well with both aggregated and interspaced strategies. However, using the aggregated strategy, a large sample size was necessary to obtain good predictions. In contrast, when the interspaced strategy was used, a sample size of only 20% was enough to get AUC values higher than 0.7 for both habitats, 0.74 sensitivity and 0.68 specificity for *L*. *byssoides* rims, and of 0.92 sensitivity and 0.81 specificity for *N*. *brassica-florida* (Fig 4, Table 3, Table C and Table D in S1 File).

For the uncommon and scattered habitat of *Hildenbrandia/Phymatholiton*, few models yielded good accuracy and performance. In fact, with the interspaced design and 20% sample size, predictions were accurate (AUC of 0.73) and specific (specificity of 0.90), but the model was not sensitive enough (sensitivity of 0.37) (Table 3). Only the aggregated case 2, with 30% sample size, provided a prediction with good accuracy and performance, possibly as a result of the higher habitat frequency (Table 3 and Table E in S1 File).

At the infralittoral level, for the widespread and abundant habitat of *Cystoseira mediterranea*, the 20% sample size interspaced model was again the most accurate, with AUC of 0.77 (Fig 4), but more sensitive (sensitivity of 0.84 and specificity of 0.61) (Table 3 and Table F in S1 File). With the exceptions of the most widespread habitats, aggregated sampling designs led to low accuracy models, independently of sample size or habitat prevalence (aggregated case 2 with 40% sample size, aggregated with 50% and interspaced with 50% sample size), (Table F in S1 File).

## Discussion

We found strong consistency (*sensu* Oreskes et al. [54]) between the distributions predicted by our models and those observed in the field for the six rocky littoral habitats studied, which ranged from uncommon to frequent and from localised to scattered along the whole coastline. Additionally, our models show that, in terms of minimum effort and highest accuracy, the interspaced is the best sampling strategy for accurate and well-performing predictions. Hirzel and Guisan [5] established that, when habitats with different distributional patterns are considered, the regular and ‘equally-stratified’ sampling strategies may yield the most accurate and robust predictive models based on simulated data. Our results from field data clearly support this idea.

Technically speaking, the interspaced sampling design ensured that the training datasets adequately represented the distribution of the environmental conditions faced by the different habitats (S1 Table). Completeness, or the degree to which the habitat spatial range of environmental variables is covered by the sample, has been shown to positively affect SDMs, especially when the SDMs are used to infer distribution data from other locations [6, 55]. Here we show that the interspaced sampling strategy reduced the environmental divergence between the two data sets better than the aggregated strategy, thus improving the accuracy of predictive models.

Sampling size has also been suggested to have strong effects on SDM or HDM predictive accuracy [5, 11, 47, 56, 57]. In our interspaced models, increasing sample size did not increase accuracy or model performance, because accuracy depends on the habitat prevalence. Thus, an interspaced sampling design also guarantees a representative coverage of habitats occurrences (prevalence) with a minimum number of observations [8]. In fact, with only 20% of the sample size (3,216 observations out of 16,098) we achieved accurate prediction models (high AUC) of the distribution of nearly all studied habitats for the rest of the coast (Fig 3). This means that by sampling a relatively small fraction of the littoral (20% of the coast), the ranges of environmental variables driving the presence or the absence of several habitats were well-covered. Our high-resolution sampling provided a large amount of high-quality observations. Thus, the split-sample approach with the interspaced design did not reduce the model capacity to fit the data [2]. However, when an aggregated strategy was used, both model accuracy and performance strongly depended on the habitat distribution. Either high sample size or high habitat prevalence in the training data set was needed to build accurate models (Figs 3 and 4). In fact, the aggregated strategy might prove useful when modelling focuses on a single habitat, but may require prior knowledge of where the habitat occurs.

**Fig 5:**
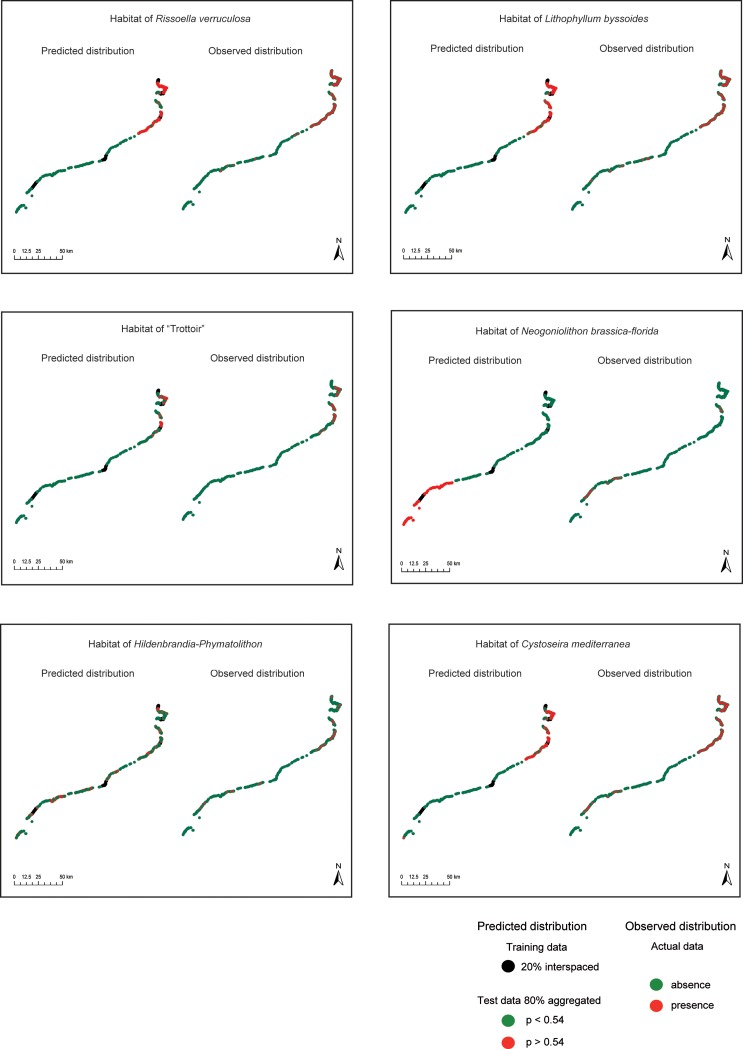
Predicted distribution vs. observed distribution along the Catalan coastline for the six habitats considered. The coastline on the left side of each panel represents the training data and the probability of habitat occurrence in the test data; the coastline in the right side represents the observed habitats as recorded in the Cartography of the Littoral Habitats (see Material and Methods section).

In order to compare predicted vs. observed distributions with the interspaced strategy and a sample size of 20%, we transformed the probabilities into binary (presence/absence) maps (Fig 5). Although all the habitats considered here contain specialist species, which are strongly associated with the environmental variables considered, we obtained the best model predictions with both abundant and uncommon habitats. This supports results obtained in previous studies [6, 58]. In fact, model effectiveness strongly depends on the relation between species and predictors [58, 59].

The habitats of *Rissoella verruculosa* and *Lithophyllum byssoides* were more abundant in the northern coast (where they occur in 68% and 49% of sampling points, respectively, Fig 5), where environmental conditions are suitable for their optimum development [34]. These habitats are spatially strongly associated with the explanatory variables used, so the accuracy of the resulting models was high. Performance was also high because the habitat prevalence in the training data reached nearly 50%, thus covering the suitable range and improving the capacity of the model to discern between presences and absences in the test data (Fig 4). Models tend to perform better when habitat prevalence is intermediate [60]. This effect is to be expected because logistic probabilities are computed on the values of the predictors as well as on the relative proportion of presence/absence data [47, 61].

For rare habitats like the *Lithophyllum byssoides* “rims” and *Neogoniolithon brassica-florida* concretions, which are uncommon but locally aggregated (present in 7.2% and 3.3% of data points, respectively, with latitudinal interquartile ranges [IQR] of 6.3 and 6.6 km) we obtained useful predictions depending on their prevalence in training data. However, the presence of *N*. *brassica-florida* was over predicted (Fig 5) although the values of both sensitivity and specificity were high.

The cave habitat dominated by *Hildenbrandia and Phymatolithon*, was uncommon but scattered along the coast [34] (0.7% of data points but latitudinal IQR of 22 km). Although highly accurate (as measured by AUC), the model showed high specificity but low sensitivity, i.e. it was able to detect habitat absence but failed to detect habitat presence (low true positive rate, Fig 5). Habitats with low prevalence in the training data and absent from many coastline points may have led to misspecification of the response curve [6, 62]. In fact, when one of the two events (presence or absence) is over represented with respect to the other, mean probabilities tend to be biased towards the most common event [47, 63–65]. The model also failed to predict the presence of the habitat of *Cystoseira mediterranea* (Fig 5). Accuracy and specific performance were moderate, although the habitat was abundant along the coast. Therefore, while model outputs were useful (as measured by AUC) they only predicted well the habitat absence but they were not the best to predict its presence.

From our results, the most important factors in model prediction were the sampling strategy and the habitat prevalence. However, we observed that low environmental dispersion between training and test data is essential to improve the outputs models. Sample size influenced the models effectiveness mostly when the aggregated strategy was used.

Our data showed that using the right sample design (interspaced) we may obtain a fair representation of habitat prevalence following the environmental variability in both our training and test datasets. Spatially biased (i.e. aggregated) survey designs have been proven to cover inefficiently the real geographic pattern of species distribution within a region [63, 64]. Some authors have stressed that incrementing sample sizes may lead to higher model performance in predicting species distributions [11]. In contrast, for uncommon habitats, either localized or scattered, increasing sample size may not necessarily increase the number of presences in the training dataset.

In brief, the interspaced sampling procedure allows reaching useful and accurate predictive models, whereas performance is dependent on the occurrence and distribution of each habitat. We also highlight that it is not only the accuracy of the model that should be considered, but performance is also crucial to get reliable ecological information on the distribution patterns. Sampling is often costly and time consuming, especially for marine environments. When the aim is to predict the geographical distributions of species and habitats, static, comparative, empirical models, rather than mechanistic models [2], may help reduce significantly the sampling effort by identifying the best sampling strategy in terms of cost and effort. This information is particularly relevant for littoral marine environments, for which SDMs have lacked so far a systematic and planned sampling strategy and model performance has never been considered. These cost effective sampling strategies can be applied to different habitats in different areas, especially those where field work and ground-truthing of habitat distributions have not been yet performed (i.e. in some unexplored areas of the southern and eastern Mediterranean Sea). Nevertheless, it is pivotal to be in possession of data about the best environmental variables to combine with habitat data, thus obtaining the best predictions across seascapes. Finally, the outcome of these models is essential to improve extensive habitat cartographies, to inform studies addressed at detecting high biodiversity areas, to identify and design protected areas and, in general, to implement management plans, especially in littoral environments.

## Supporting information

S1 File **Tables A-F. Results of logistic regression models.** Results of logistic regression models for all sampling strategy designs are presented for each habitat and for all sample sizes. For training data, the number (N) and frequency (F) of the habitat occurrence are presented. Results of null models are shown with the mead and standard deviation of the 10 models calculated. The D^2^ is the Deviance of the model in the training data; AUC is the area under the receiver operating characteristic (ROC) curve, se and spe are the sensitivity and specificity respectively, for the predictive model in the test data.
(PDF)

S1 Table Full data base. Projected coordinates, environmental variables and the presence/absence (1/0) of each habitat are presented for each point. Slope code: 1 = 0º-10.8º; 2 = 10.8º-22.8º; 3 = 22.8º-45.1º; 4 = 45.1º-68.2º; 5 = 68.2º-87.8º. Habitats code: Riv = *Rissoella verruculosa*; Lby = *Lithophyllum byssoides*; Tro = *Lithophyllum byssoides* rims ("Trottoir”); Neo = *Neogoniolithon brassica-florida*; Hph = *Hildenbrandia rubra/ Phymatolithon lenormandi*.
(XLSX)
