# Supplementary material for: The optimal sampling design for littoral habitats modelling: A case study from the north-western Mediterranean
Source: PLoS One. 2018 May 24;13(5):e0197234. doi: 10.1371/journal.pone.0197234 (PMC5967749; doi:10.1371/journal.pone.0197234)
Supplement: S1 File — Tables A-F. Results of logistic regression models. Results of logistic regression models for all sampling strategy designs are presented for each habitat and for all sample sizes. For training data, the number (N) and frequency (F) of the habitat occurrence are presented. Results of null models are shown with the mead and standard deviation of the 10 models calculated. The D2 is the Deviance of the model in the training data; AUC is the area under the receiver operating characteristic (ROC) curve, se and spe are the sensitivity and specificity respectively, for the predictive model in the test data. (PDF) [file pone.0197234.s001.pdf]

S1 File

Tables A-F. Results of logistic regression models

Results of logistic regression models for all sampling strategy designs are presented for each habitat and for all sample sizes. For training data, the number (N) and frequency (F) of the habitat occurrence are presented. Results of null models are shown with the mead and standard deviation of the 10 models calculated. The D^2 is the Deviance of the model in the training data; AUC is the area under the receiver operating characteristic (ROC) curve, se and spe are the sensitivity and specificity respectively, for the predictive model in the test data.

Table A. Habitat of *Rissoella verruculosa*

Models strategy	training data	N	F	D2	test data	AUC	threshold	se	spe
**Aggregated**									
	10%	686	0,4261	34,98	90%	0,62	0,55	0,98	0,35
	20%	1039	0,3226	53,70	80%	0,71	0,28	0,45	0,89
	30%	1308	0,2935	19,39	70%	0,71	0,20	0,62	0,67
	40%	1803	0,2780	19,39	60%	0,66	0,43	0,46	0,77
	50%	2509	0,3123	62,20	50%	0,68	0,48	0,82	0,47
**Interspaced**									
	10%	146	0,0900	58	90%	0,82	0,02	0,96	0,44
	20%	1614	0,4936	49	80%	0,87	0,57	0,90	0,74
	30%	1545	0,3219	56,30	70%	0,86	0,71	0,81	0,76
	40%	2751	0,4273	51	60%	0,86	0,37	0,92	0,70
	50%	3610	0,4500	51,90	50%	0,86	0,02	1,00	0,63
**Aggregated case 1**									
	10%	319	0,2000	14	90%	0,46	0,67	0,00	1,00
	20%	2467	0,7659	9,15	80%	0,76	0,07	0,23	0,90
	30%	3857	0,7569	8,90	70%	0,86	0,12	0,97	0,74
	40%	3774	0,5821	24	60%	0,94	0,01	0,72	0,91
**Aggregated case 2**									
	10%	466	0,2900	36,00	90%	0,35	0,01	0,92	0,18
	20%	2084	0,6375	42,00	80%	0,18	0	1	0
	30%	3915	0,8206	6,20	70%	0,88	0,69	0,98	0,69
	40%	4043	0,6283	33,40	60%	0,90	0,17	0,98	0,74
**Aggregated case 3**									
	10%	75	0,0500	NA	90%	NA	NA	NA	NA
	20%	15	0,0047	6	80%	0,72	0,05	0,80	0,59
	30%	15	0,0036	10,40	70%	0,61	0,99	0,88	0,28
	40%	1283	0,1998	73	60%	0,74	0,53	0,61	0,86
**Aggregated case 4**									
	50%_a	5125	0,6368	16,60	50%_b	0,94	0,44	0,96	0,91
	50%_b	2584	0,3210	67,10	50%_a	0,73	0,88	0,71	0,71
**Null models**									
mean	10%	761	0,4726	45,84	90%	0,89	0,45	0,89	0,77
sd		18	0,011	2,22		0,00	0,04	0,03	0,02
mean	20%	1532	0,4762	45,00	80%	0,89	0,45	0,90	0,77
sd		32	0,010	0,79		0,00	0,02	0,02	0,01
mean	30%	2306	0,4779	44,65	70%	0,89	0,45	0,90	0,77
sd		34	0,007	1,42		0,00	0,01	0,01	0,01
mean	40%	3056	0,4751	44,35	60%	0,89	0,45	0,91	0,77
sd		45	0,007	0,85		0,00	0,02	0,01	0,01
mean	50%	3839	0,4774	44,66	50%	0,89	0,46	0,90	0,78
sd		32	0,004	0,62		0,00	0,01	0,01	0,00

Table B. Habitat of *Lithophyllum byssoides*

Models strategy

	training data	N	F	D2	test data	AUC	threshold	se	spe
**Aggregated**									
	10%	658	0,4087	29,55	90%	0,80	0,05	0,53	0,82
	20%	1123	0,3486	53,70	80%	0,75	0,43	0,97	0,52
	30%	1343	0,3014	27,84	70%	0,68	0,29	0,97	0,43
	40%	1828	0,2818	19,39	60%	0,68	0,58	0,95	0,38
	50%	2505	0,3118	62,20	50%	0,74	0,59	0,85	0,50
**Interspaced**									
	10%	443	0,2700	39	90%	0,74	0,02	0,96	0,46
	20%	1304	0,3988	25,70	80%	0,77	0,40	0,92	0,54
	30%	1016	0,2421	27,60	70%	0,75	0,24	0,96	0,42
	40%	1800	0,2796	29,50	60%	0,75	0,30	0,90	0,51
	50%	2844	0,3500	25,60	50%	0,76	0,19	0,94	0,48
**Aggregated case 1**									
	10%	867	0,5400	5	90%	0,30	0,47	0,99	0,017
	20%	1865	0,5790	4,40	80%	0,72	0,09	0,25	0,89
	30%	2691	0,5281	5,30	70%	0,80	0,12	0,93	0,62
	40%	2951	0,4552	8,80	60%	0,85	0,01	0,81	0,77
**Aggregated case 2**									
	10%	571	0,3500	16	90%	0,26	0	1	0
	20%	1575	0,4818	7,80	80%	0,27	0,09	0,98	0,03
	30%	2551	0,5347	3,30	70%	0,50	0,36	0,90	0,62
	40%	2784	0,4326	13	60%	0,83	0,13	0,95	0,62
**Aggregated case 3**									
	10%	18	0,0100	NA	90%	NA	NA	NA	NA
	20%	119	0,0370	16,20	80%	0,74	0,93	0,96	0,45
	30%	109	0,0227	23,50	70%	0,58	0,97	0,91	0,19
	40%	932	0,1451	7,30	60%	0,50	0,53	0,97	0,08
**Aggregated case 4**									
	50%_a	3917	0,4867	5,70	50%_b	0,87	0,33	0,89	0,77
	50%_b	1703	0,2116	36	50%_a	0,53	0,62	0,63	0,45
**Null models**									
mean	10%	554	0,3439	24,31	90%	0,79	0,23	0,96	0,50
sd		16	0,0102	1,61		0,002	0,03	0,005	0,005
mean	20%	1111	0,3453	23,70	80%	0,79	0,25	0,96	0,51
sd		37	0,0114	1,02		0,001	0,08	0,302	0,161
mean	30%	1689	0,3499	23,67	70%	0,79	0,24	0,96	0,50
sd		29	0,0060	0,61		0,002	0,02	0,002	0,003
mean	40%	2221	0,3453	23,33	60%	0,79	0,24	0,96	0,51
sd		42	0,0065	0,71		0,004	0,05	0,016	0,019
mean	50%	2796	0,3476	23,45	50%	0,79	0,24	0,96	0,50
sd		25	0,0031	0,30		0,002	0,08	0,304	0,160

Table C. Habitat of *Lithophyllum byssoides* rim ("Trottoir")

Models strategy	training data	N	F	D2	test data	AUC	threshold	se	spe
**Aggregated**									
	10%	15	0,01	34,98	90%	0,69	0,90	0,53	0,82
	20%	17	0,01	53,70	80%	0,66	0,04	0,05	0,96
	30%	17	0,00	29,03	70%	0,63	0,03	0,00	1,00
	40%	51	0,01	19,39	60%	0,46	0,00	1	0
	50%	175	0,02	62,20	50%	0,66	0,15	0,86	0,40
**Interspaced**									
	10%	267	0,160	35	90%	0,87	0,18	0,74	0,86
	20%	102	0,031	27	80%	0,75	0,03	0,74	0,68
	30%	282	0,059	27	70%	0,81	0,07	0,82	0,68
	40%	268	0,042	33	60%	0,79	0,05	0,72	0,81
	50%	931	0,116	37	50%	0,83	0,04	0,79	0,84
**Aggregated case 1**									
	10%	52	0,030	66	90%	0,43	0,01	0,02	0,98
	20%	160	0,050	23	80%	0,38	1	0	1
	30%	183	0,036	17	70%	0,69	0,01	0,74	0,61
	40%	942	0,145	41	60%	0,90	0,04	0,88	0,79
**Aggregated case 2**									
	10%	749	0,460	28	90%	0,73	0,02	0,94	0,58
	20%	786	0,240	37	80%	0,63	0,49	0,95	0,48
	30%	212	0,044	20	70%	0,66	0,001	1	0,31
	40%	859	0,133	46	60%	0,60	0,01	1	0,32
**Aggregated case 3**									
	10%	0	0,000	NA	90%	NA	NA	NA	NA
	20%	0	0,000	NA	80%	NA	NA	NA	NA
	30%	0	0,000	NA	70%	NA	NA	NA	NA
	40%	16	0,002	22	60%	0,82	0,18	0,68	0,86
**Aggregated case 4**									
	50%_a	1133	0,141	22	50%_b	0,37	0,28	0,71	0,38
	50%_b	23	0,003	22	50%_a	0,82	0,18	0,75	0,79
**Null models**									
mean	10%	112,4	0,0698	32,74	90%	0,89	0,08	0,82	0,81
sd		7,7	0,0048	2,87		0,002	0,02	0,037	0,040
mean	20%	228,6	0,0711	32,60	80%	0,89	0,08	0,82	0,80
sd		10,7	0,0033	2,06		0,003	0,02	0,041	0,038
mean	30%	344,7	0,0714	31,70	70%	0,89	0,07	0,83	0,79
sd		18,2	0,0038	1,79		0,005	0,02	0,038	0,035
mean	40%	460,2	0,0715	31,68	60%	0,89	0,07	0,84	0,78
sd		11,3	0,0018	1,59		0,006	0,03	0,042	0,044
mean	50%	577,4	0,0718	31,82	50%	0,89	0,07	0,84	0,78
sd		21,1	0,0026	0,76		0,004	0,02	0,040	0,040

Table D. Habitat of *Neogoniolithon brassica-florida*

Models strategy	training data	N	F	D2	test data	AUC	threshold	se	spe
**Aggregated**									
	10%	0	0	34,98	90%	0,19	0	1	0
	20%	0	0	53,70	80%	0,72	0	1	0
	30%	24	0,01	29,03	70%	0,90	0,21	0,94	0,87
	40%	157	0,02	19,39	60%	0,91	0,21	0,94	0,89
	50%	422	0,05	62,20	50%	0,85	0,31	0,85	0,90
**Interspaced**									
	10%	443	0,270	39	90%	0,74	0,02	0,96	0,46
	20%	1304	0,399	25,70	80%	0,77	0,4	0,92	0,54
	30%	1016	0,242	27,60	70%	0,75	0,24	0,96	0,42
	40%	1800	0,280	29,50	60%	0,75	0,3	0,90	0,51
	50%	2844	0,350	25,60	50%	0,76	0,19	0,94	0,48
**Aggregated case 1**									
	10%	867	0,540	5	90%	0,3	0,47	0,99	0,017
	20%	1865	0,579	4,40	80%	0,72	0,09	0,25	0,89
	30%	2691	0,528	5,30	70%	0,80	0,12	0,93	0,62
	40%	2951	0,455	8,80	60%	0,85	0,01	0,81	0,77
**Aggregated case 2**									
	10%	571	0,350	16	90%	0,26	0	1	0
	20%	1575	0,482	7,80	80%	0,27	0,09	0,98	0,03
	30%	2551	0,535	3,30	70%	0,50	0,36	0,90	0,62
	40%	2784	0,433	13	60%	0,83	0,13	0,95	0,62
**Aggregated case 3**									
	10%	18	0,010	NA	90%	NA	NA	NA	NA
	20%	119	0,037	16,20	80%	0,74	0,93	0,96	0,45
	30%	109	0,023	23,50	70%	0,58	0,97	0,91	0,19
	40%	932	0,145	7,30	60%	0,50	0,53	0,97	0,08
**Aggregated case 4**									
	50%_a	3917	0,487	5,70	50%_b	0,87	0,33	0,89427	0,77401
	50%_b	1703	0,212	36,20	50%_a	0,53	0,62	0,63441	0,44783
**Null models**									
mean	10%	553,7	0,344	24,31	90%	0,79	0,23	0,96	0,50
sd		16,4	0,010	1,61		0,002	0,03	0,005	0,005
mean	20%	1110,9	0,345	23,70	80%	0,79	0,25	0,96	0,51
sd		36,6	0,011	1,02		0,001	0,08	0,302	0,161
mean	30%	1688,5	0,350	23,67	70%	0,79	0,24	0,96	0,50
sd		28,8	0,006	0,61		0,002	0,02	0,002	0,003
mean	40%	2221,2	0,345	23,33	60%	0,79	0,24	0,96	0,51
sd		41,6	0,006	0,71		0,004	0,05	0,016	0,019
mean	50%	2795,5	0,348	23,45	50%	0,79	0,24	0,96	0,50
sd		24,7	0,003	0,30		0,002	0,08	0,304	0,160

Table E. Habitat of *Hildenbrandia - Phymatholiton*

Models strategy	training data	N	F	D2	test data	AUC	threshold	se	spe
**Aggregated**									
	10%	20	0,012	34,98	90%	0,64	0,01	0,59	0,72
	20%	52	0,016	53,70	80%	0,65	0,01	0,90	0,41
	30%	63	0,014	19,39	70%	0,63	0,01	1	0,27
	40%	76	0,012	19,39	60%	0,76	0,02	0,61	0,79
	50%	92	0,011	62,20	50%	0,66	0,01	0,4	0,83
**Interspaced**									
	10%	5	0,0030	NA	90%	NA	NA	NA	NA
	20%	12	0,0037	20,40	80%	0,73	0,01	0,37	0,90
	30%	16	0,0033	13,40	70%	0,81	0,01	0,65	0,79
	40%	72	0,0112	12	60%	0,64	0,01	0,68	0,59
	50%	75	0,0093	8	50%	0,67	0,03	0,48	0,89
**Aggregated case 1**									
	10%	2	0,0010	NA	90%	NA	NA	NA	NA
	20%	6	0,0019	NA	80%	NA	NA	NA	NA
	30%	10	0,0020	NA	70%	NA	NA	NA	NA
	40%	25	0,0039	9,80	60%	0,51	0,84	0,12	0,95
**Aggregated case 2**									
	10%	9	0,0050	NA	90%	NA	NA	NA	NA
	20%	31	0,0095	8,70	80%	0,68	0,01	0,73	0,56
	30%	77	0,0161	12,20	70%	0,82	0,02	0,74	0,81
	40%	84	0,0131	9,20	60%	0,71	0,03	0,46	0,92
**Aggregated case 3**									
	10%	2	0,0010	NA	90%	NA	NA	NA	NA
	20%	17	0,0053	8,70	80%	0,72	0,01	0,67	0,67
	30%	17	0,0041	26	70%	0,47	0,99	0,53	0,52
	40%	49	0,0076	12,20	60%	0,45	0,06	0,87	0,22
**Aggregated case 4**									
	50%_a	41	0,0093	8,90	50%_b	0,50	0,02	0,80	0,34
	50%_b	78	0,0055	14,10	50%_a	0,64	0,12	0,63	0,70
**Null models**									
mean	10%	11,1	0,0069	23,24	90%	0,78	0,01	0,56	0,82
sd		2,0	0,0013	8,97		0,029	0,004	0,12	0,06
mean	20%	21,6	0,0067	18,37	80%	0,79	0,01	0,56	0,84
sd		2,1	0,0006	3,17		0,031	0,004	0,06	0,03
mean	30%	33,8	0,0070	16,60	70%	0,80	0,01	0,59	0,83
sd		3,4	0,0007	2,72		0,029	0,004	0,09	0,04
mean	40%	43,8	0,0068	16,19	60%	0,80	0,01	0,60	0,83
sd		5,4	0,0008	1,56		0,022	0,000	0,05	0,03
mean	50%	59,3	0,0074	15,76	50%	0,81	0,01	0,61	0,82
sd		6,6	0,0008	2,60		0,026	0,003	0,07	0,04

Table F. Habitat of *Cystoseira mediterranea*

Models strategy	training data	N	F	D2	test data	AUC	threshold	se	spe
**Aggregated**									
	10%	686	0,426	34,98	90%	0,62	0,55	0,98	0,35
	20%	1039	0,323	53,70	80%	0,71	0,28	0,45	0,89
	30%	1308	0,294	19,39	70%	0,71	0,20	0,62	0,67
	40%	1803	0,278	19,39	60%	0,66	0,43	0,46	0,77
	50%	2509	0,312	62,20	50%	0,68	0,48	0,82	0,47
**Interspaced**									
	10%	149	0,090	20	90%	0,69	0,02	0,96	0,44
	20%	966	0,295	16	80%	0,77	0,37	0,84	0,61
	30%	899	0,187	27,50	70%	0,50	0,01	0,89	0,15
	40%	1585	0,246	16,40	60%	0,65	0,24	0,76	0,47
	50%	1869	0,230	20	50%	0,77	0,19	0,87	0,58
**Aggregated case 1**									
	10%	319	0,200	16	90%	0,56	0,67	0,00	1,00
	20%	990	0,307	5,30	80%	0,32	0,19	0,99	0,01
	30%	1736	0,341	8,90	70%	0,31	0,91	0,92	0,14
	40%	1976	0,305	5,90	60%	0,69	0,10	1,00	0,45
**Aggregated case 2**									
	10%	466	0,290	14	90%	0,50	0,01	0,92	0,18
	20%	1421	0,435	9,70	80%	0,42	0,07	0,77	0,28
	30%	2414	0,506	8,60	70%	0,68	0,19	0,98	0,37
	40%	2615	0,406	9,40	60%	0,71	0,31	0,67	0,65
**Aggregated case 3**									
	10%	75	0,05	33	90%	0,49	0,00	1,00	0,00
	20%	179	0,06	17,30	80%	0,31	0,00	1,00	0,00
	30%	186	0,04	15,40	70%	0,42	0,00	1,00	0,00
	40%	1037	0,16	31,30	60%	0,48	0,49	0,98	0,05
**Aggregated case 4**									
	50%_a	2778	0,35	6,30	50%_b	0,60	0,43	1,00	0,44
	50%_b	1797	0,22	38	50%_a	0,62	0,38	0,56	0,60
**Null models**									
mean	10%	459,3	0,2853	21,67	90%	0,78	0,29	0,84	0,59
sd		18,4	0,0114	1,15		0,003	0,016	0,025	0,025
mean	20%	919,3	0,2858	21,49	80%	0,78	0,28	0,85	0,59
sd		29,5	0,0092	0,80		0,002	0,024	0,016	0,017
mean	30%	1363,7	0,2826	21,07	70%	0,78	0,28	0,85	0,59
sd		25,0	0,0052	0,70		0,002	0,015	0,016	0,017
mean	40%	1826,5	0,2839	21,04	60%	0,78	0,29	0,83	0,60
sd		34,7	0,0054	0,50		0,003	0,024	0,030	0,029
mean	50%	2277,4	0,2832	20,89	50%	0,78	0,28	0,84	0,59
sd		26,8	0,0033	0,37		0,003	0,032	0,037	0,037
